# Effects of Bilateral Transcranial Direct Current Stimulation on Simultaneous Bimanual Handgrip Strength

**DOI:** 10.3389/fnhum.2021.674851

**Published:** 2021-06-02

**Authors:** Mikito Hikosaka, Yu Aramaki

**Affiliations:** ^1^Graduate School of Health and Sport Sciences, Chukyo University, Aichi, Japan; ^2^School of Health and Sport Sciences, Chukyo University, Aichi, Japan

**Keywords:** transcranial direct current stimulation, handgrip strength, unimanual movement, bimanual movement, bilateral deficit

## Abstract

Although the effects of transcranial direct current stimulation (tDCS) on contralateral unimanual movement have been well reported, its effects on coordinated multi-limb movements remain unclear. Because multi-limb coordination is often performed in daily activities and sports, clarifying the effects of tDCS on multi-limb coordination may have valuable implications. However, considering the neural crosstalk involved in bimanual movements, including the transcallosal pathway and ipsilateral motor pathway, the extent of tDCS-induced improvement may differ between unimanual and bimanual movement. We examined how tDCS affects simultaneous bimanual maximal voluntary contraction (MVC) by testing the effects of tDCS of the bilateral primary motor cortex (M1) on unimanual and bimanual handgrip strength. Twenty-one right-handed healthy adults underwent three bilateral tDCS protocols (“RaLc,” with an anode on right M1 and a cathode on left M1, “RcLa,” with an anode on left M1 and a cathode on right M1, and “Sham”) in a randomized order. A 1.5 mA current was applied for 15 min during tDCS. Participants then performed maximal unimanual and bimanual handgrip tests. Bimanual handgrip force was higher in both hands in the RcLa condition than in the Sham condition. Similarly, unimanual handgrip force was higher in the RcLa condition than in the Sham condition. Stimulus responses were asymmetrical and were not observed in the RaLc condition. Our findings demonstrate that RcLa tDCS leads to neuromodulation that can produce greater unimanual and bimanual handgrip strength. This result provides basic evidence that tDCS may be useful in sports, particularly those involving bilateral coordination of upper limb movement.

## Introduction

Electrical brain stimulation has received substantial interest in sports science as a tool for enhancing sports performance (Colzato et al., 2016; Reardon, 2016; Edwards et al., 2017). Regardless of whether this technology constitutes doping, its application to sports warrants thorough examination with primary research. Previous studies reported that transcranial direct current stimulation (tDCS) improves unimanual motor performance of the contralateral upper limbs (Cogiamanian et al., 2007; Krishnan et al., 2014). These findings may be useful for neurorehabilitation, including movement recovery in paralyzed hands. However, it remains unclear whether tDCS is directly related to improving sports performance because most sports involve coordinated multi-limb movement. In particular, activity in the transcallosal pathway (Kennerley et al., 2002) and ipsilateral motor pathway (Kagerer et al., 2003) have been reported to cause bilateral interactions (Swinnen, 2002; Carson, 2005) in sports involving bilateral coordination of upper limb movement, such as rowing and weightlifting. Therefore, it is difficult to predict how modifying these complex neural mechanisms with tDCS will affect sports performance. Thus, we conducted a basic study of the application of tDCS to sports, examining how tDCS affects unimanual and simultaneous bimanual motor performance.

tDCS has traditionally been considered to induce neuromodulation, with anodal stimulation increasing contralateral corticospinal excitability, and cathodal stimulation decreasing it in a polarity-dependent manner (Nitsche and Paulus, 2000). Previous studies focused on improving contralateral unimanual movement by anodal tDCS of the primary motor cortex (M1; Cogiamanian et al., 2007; Krishnan et al., 2014), which suggests that stimulation facilitated the contralateral motor pathway. In particular, Krishnan et al. (2014) reported that anodal tDCS of left M1 increased unimanual force-generating capacity and muscle activity of the right elbow flexor and extensor. However, because most tDCS studies only examined unimanual movement, the effects of tDCS on bimanual movement are not well known.

Force generated in the simultaneous bimanual use of two limbs causes reduced performance of each limb, known as bilateral deficit (BLD; Henry and Smith, 1961; Ohtsuki, 1981; Škarabot et al., 2016). Henry and Smith (1961) reported a 3% reduction in bimanual right handgrip strength compared with unimanual right handgrip. Interhemispheric inhibition (IHI) in the transcallosal pathway is a major neurophysiological factor in BLD (Oda and Moritani, 1995; Perez et al., 2014; Škarabot et al., 2016). Meanwhile, cross-activation has also been reported, in which the corticospinal excitability of the non-exercise side increases with unimanual force generation (Hortobagyi et al., 2003; Perez and Cohen, 2008). Further, most corticospinal fibers cross the pyramidal decussation, whereas some fibers do not cross and descend to the ipsilateral spinal cord (Ziemann et al., 1999; Ziemann and Hallett, 2001; Lacroix et al., 2004). In addition, previous studies reported that monkey M1 contains motor neurons that are active during bimanual movement (Aizawa et al., 1990; Donchin et al., 1998). Because neural crosstalk is involved in bimanual movement, as mentioned above, the effects of tDCS on bimanual movement may differ from the effects on unimanual movement.

Therefore, we hypothesized that tDCS, which increases unimanual movements does not necessarily increase bimanual movements to the same extent. In other words, we predicted that the improvement in bimanual movement induced by tDCS may be larger or smaller than the improvement in unimanual movement. Thus, to assess how tDCS affects simultaneous bimanual force generation, we tested the effects of tDCS of bilateral M1 on unimanual and bimanual handgrip strength.

## Materials and Methods

### Participants

Twenty-one healthy adult men participated in this study (21.7 ± 0.8 years). We estimated the sample size using PANGEA v0.2 (Westfall, 2016) for a three fixed effects design, including stimulation (RaLc, RcLa, and Sham), hand (left and right), and mode (unimanual and bimanual), with a power of 0.8, the effect size of 0.5, and replicates of 2. Participants were right-handed and scored between 60 and 100 on the Edinburgh Handedness test (Oldfield, 1971). No participants had a history of neurological or psychiatric disorders, musculoskeletal injury, or neuromuscular disease, and none had undergone specific training of the hands or arms. Participants gave written informed consent in accordance with the Declaration of Helsinki. The experimental protocol was approved by the Human Subjects Committee at Chukyo University Graduate School of Health and Sport Sciences.

### Experimental Design

Participants visited the laboratory four times, undergoing one familiarization session and three experimental sessions. In the familiarization session, participants received a description of the experiment and practiced handgrip maximal voluntary contraction (MVC). The experimental sessions employed a double-blind, sham-controlled, crossover design. Randomization was performed by random number generation. To exclude fatigue of the experiment and any carryover effect of tDCS, experimental sessions were separated by >72 h. There was typically an interval of 3–10 days. All experimental sessions were performed in the same time slot to minimize daily variability. Participants were instructed to avoid alcohol for 24 h, and to avoid caffeine, medication, and strenuous exercise for 12 h before each session.

**Figure 1:**
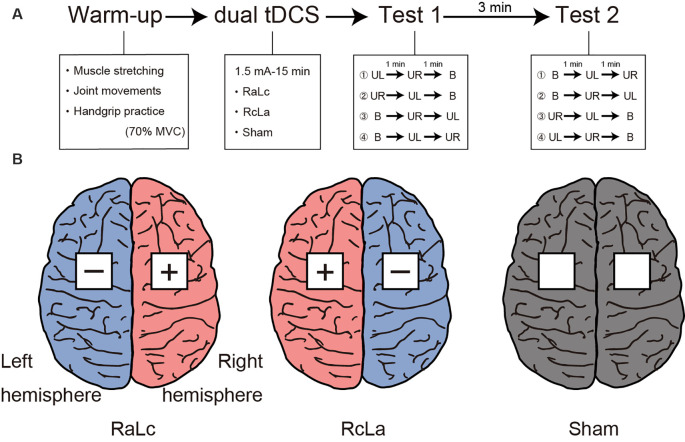
**(A)** Experimental procedure. Participants first performed a warm-up with muscle stretching, joint movements, and handgrip practice. Subsequently, participants received each bilateral transcranial direct current stimulation (tDCS) applied to the bilateral primary motor cortex (M1) for 15 min. Finally, participants performed Test 1 and Test 2 to measure handgrip strength. MVC, maximal voluntary contraction; UL, unimanual left handgrip; UR, unimanual right handgrip; B, bimanual handgrip. **(B)** Each tDCS montage left, RaLc, the anode on the right M1 and the cathode on the left M1; middle, RcLa, the anode on the left M1 and cathode on the right M1; right, Sham condition.

Figure 1A shows the experimental procedure. The experimental session consisted of a warm-up, tDCS, and two handgrip MVC tests. Participants first warmed up with muscle stretching, joint movements, and handgrip practice. Specifically, participants performed stretching of the wrist flexor/extensor muscles for 10 s, and wrist flexion/extension and internal/external rotation and finger flexion/extension 10 times, then submaximal unimanual left and right and bimanual handgrips at 70% of MVC. Next, tDCS was applied for 15 min (see “Transcranial Direct Current Stimulation” section). After tDCS, participants performed handgrip MVC tests. The test was counterbalanced to minimize the order effects of the unimanual and bimanual modes. For Test 1, participants performed unimanual left (or right) handgrip and unimanual right (or left) handgrip, then bimanual handgrip. Each task interval lasted 1 min. After 3 min of Test 1, participants performed Test 2. For Test 2, participants performed bimanual handgrip, then unimanual left (or right) handgrip, and unimanual right (or left) handgrip. Maximal handgrip was sustained for 5 s. Participants were instructed to grip as quickly and strongly as possible when prompted by the instruction “Ready? Go,” and to sustain maximal force for 5 s until the instruction “Stop” was presented. During the test, participants were seated with their shoulders adducted and neutrally rotated, elbows flexed at 90°, forearms in a neutral position, and wrists between 0 and 30° dorsiflexion, and between 0° and 15° ulnar deviation. Participants were instructed to move as little as possible.

### Transcranial Direct Current Stimulation

tDCS was delivered using a battery-driven DC Stimulator Plus (NeuroConn, Ilmenau, Germany) with a pair of 5 × 5 cm sponge surface electrodes soaked in 0.9% NaCl. The tDCS parameters were based on those reported in a previous study (Furuya et al., 2014; Tazoe et al., 2014). Because previous neuroimaging studies confirmed the presence of M1 activity at C3 and C4 in the international 10-20 system (Okamoto et al., 2004), the electrodes were placed over scalp coordinates C3 and C4 as bilateral M1. The three stimulation conditions were the anode on the right M1 (C4) and the cathode on the left M1 (C3) “RaLc,” anode on the left M1 (C3) and cathode on the right M1 (C4) “RcLa,” and “Sham” (electrodes on C3 and C4 as control; Figure 1B). A 1.5 mA current was applied for 15 min during bilateral tDCS (ramping time: 30 s). These stimulation parameters have been reported to increase contralateral corticospinal excitability on the anodal side and decrease it on the cathodal side (Tazoe et al., 2014). For the Sham condition, the current was turned off after 30 s. During stimulation, participants sat in a relaxed position and were instructed to avoid thinking of anything specific. At the end of the session, participants reported any discomfort and were asked “Do you believe that you received a real or placebo stimulation?” based on the International Federation of Clinical Neurophysiology guidelines (Antal et al., 2017).

### Data Acquisition and Processing

Handgrip force was sampled at 1,000 Hz using a grip force transducer (MLT004/ST, AD Instruments) and a data acquisition device (PL3516, AD Instruments). Sampled data were smoothed by an online low-pass filter with a cut-off frequency of 20 Hz using LabChart software (LabChart 8, AD Instruments). Maximal handgrip force was determined as the highest value. Furthermore, the maximal handgrip force in Test 1 and 2 were averaged and the BLD was calculated using the following equation.

$$bilateral\;deficit\;(\%)\;=\;\frac{bimanual\;-\;unimanual}{unimanual}\;\times \;100.$$

Surface electromyography (EMG) signals were recorded from the flexor digitorum superficialis using a wireless EMG sensor (pico, cometa). On the basis of a previous study (Kong et al., 2010), disposable Ag/AgCl electrodes were placed slightly ulnarly on the line between the oblique line of radius and the second middle phalanx at 1/4 from the oblique line of the radius. The signals were sampled at 1,000 Hz using a data acquisition device (PL3516, AD Instruments) and filtered with a band-pass filter (10–500 Hz). Recorded data were rectified and smoothed with a fourth order zero-lag Butterworth low-pass filter with a cut-off frequency of 10 Hz. Integrated EMG (IEMG) was calculated as 125 ms around the maximal handgrip force.

### Statistical Analysis

We conducted linear mixed-effects models (LMEM) analysis for maximal handgrip force and IEMG using R and the lme4 software package (Bates et al., 2015). LMEM can consider learning or fatigue effects and chronometric paradigms (Baayen et al., 2008). We performed maximal LMEM analysis based on previous studies (Barr et al., 2013; Brauer and Curtin, 2018). As a fixed effect, a total of 12 stimulation conditions (three levels: RaLc vs. RcLa vs. Sham), hand (two levels: left vs. right), and mode (two levels: unimanual vs. bimanual) and their interactions were included in this model. In addition, 12 by-subject random effects associated with these fixed effects were included in the model. Furthermore, the total of eight by-subject random intercepts and slopes for experiment date (Date 1, 2, and 3), test (Test 1 and 2), and handgrip order (Order 1, 2, and 3) were included in the model. For IEMG analysis, random effects in test and handgrip order were excluded because maximal LMEM did not converge.

We then analyzed *F*- and *p*-values for interactions and main effects using Type III analysis of variance (ANOVA) with Satterthwaite’s degrees of freedom approximation using lmerTest package (Kuznetsova et al., 2017). In addition, *t*- and *p*-values between the levels of stimulation condition were calculated and p-values were adjusted using the Benjamini–Hochberg (BH) method (Benjamini and Hochberg, 1995). When interactions and main effects related to stimulation condition were significant, a *post hoc* test on a subset of contrasts between the handgrip tasks was performed on the estimates, and *t*- and *p*-values were calculated using the emmeans package (Lenth, 2020) based on the hypothesis that the effects of tDCS on unimanual and bimanual handgrip strength would be different. *P*-values were adjusted using the BH method (Benjamini and Hochberg, 1995). Effect sizes for estimates were computed by dividing the difference between estimated means by the square root of the sums of the variance of the random parameters (Westfall et al., 2014; Judd et al., 2017).

Each BLD was compared with zero using one-sample *t*-test (one-tailed) in SPSS version 26 (IBM SPSS statistics) and the effect sizes were computed by dividing the mean difference by the standard deviation. Adverse effects and placebo effects were analyzed using the Friedman test. All data were reported as observed mean ± standard deviation (SD). Statistical significance was defined as α < 0.05.

## Results

### Linear Mixed-Effects Model Analysis of Maximal Handgrip Force and IEMG

Table 1 shows the results of LMEM analysis of maximal handgrip force and IEMG. Regarding maximal handgrip force, Type III ANOVA revealed no significant interactions between fixed effects (all *p*-values > 0.05; Table 1). However, there were significant main effects of stimulation condition (Stimulation: *F*_(2,14.3)_ = 10.295, *p* = 0.002; Hand: *F*_(1,20.0)_ = 12.164, *p* = 0.002; Mode: *F*_(1,20.5)_ = 12.917, *p* = 0.002; Table 1). In the stimulation conditions, maximal handgrip force was higher in the RcLa condition than in the Sham condition (*t*_(16)_ = 4.304, *p* = 0.002, *d* = 0.11). Meanwhile, higher handgrip force was not observed in the RaLc condition compared with that in the Sham condition (*t*_(21)_ = 1.394, *p* = 0.178, *d* = 0.06). However, no significant differences were observed between the RcLa and RaLc conditions (*t*_(13)_ = 1.442, *p* = 0.178, *d* = 0.05). For IEMG, Type III ANOVA revealed no significant interactions between the fixed effects in IEMG (all *p*-values > 0.05; Table 1). A significant main effect was observed only for hand (*F*_(1,20.0)_ = 11.953, *p* = 0.002).

**Table 1:** Summary of linear mixed-effects model analysis of variance.

	Effect	*F*-value	dfN	dfD	*p*-value
Handgrip force	Stim	10.295	2	14.3	0.002
	Hand	12.164	1	20.0	0.002
	Mode	12.917	1	20.5	0.002
	Stim×Hand	0.708	2	24.8	0.502
	Stim×Mode	0.048	2	21.1	0.953
	Hand×Mode	1.908	1	22.5	0.181
	Stim×Hand×Mode	0.405	2	27.8	0.671
IEMG	Stim	0.159	2	19.9	0.854
	Hand	11.953	1	20.0	0.002
	Mode	0.075	1	26.2	0.786
	Stim×Hand	1.598	2	20.3	0.227
	Stim×Mode	0.008	2	29.0	0.992
	Hand×Mode	0.018	1	71.4	0.894
	Stim×Hand×Mode	0.344	2	35.9	0.711

*IEMG, integrated electromyography; Stim, stimulation condition; dfN, degrees of freedom numerator; dfD, degrees of freedom denominator*.

**Figure 2:**
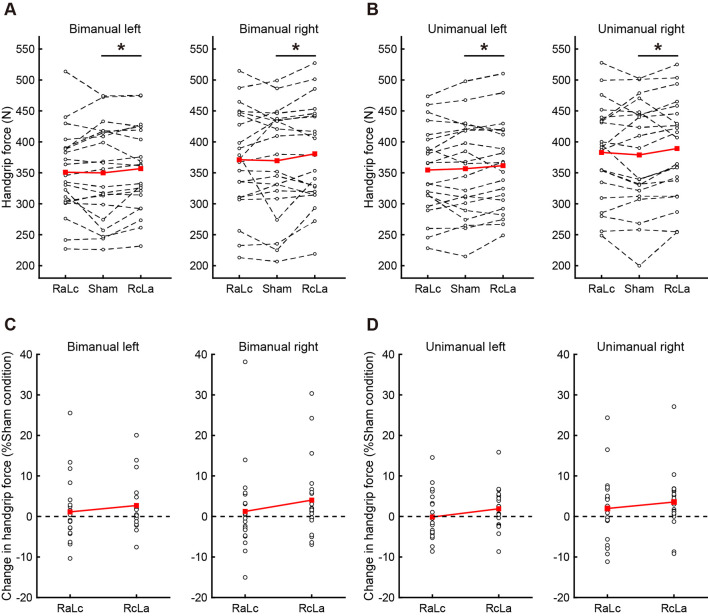
The top figures indicate individual maximal handgrip force for **(A)** bimanual and **(B)** unimanual tasks. White circles and dashed lines show individual data, while red squares and solid lines show the averaged data. The bottom figures indicate the change in **(C)** bimanual and **(D)** unimanual handgrip strength relative to the Sham condition, and positive values indicate greater handgrip strength. White circles show individual data, while red squares and solid lines show the averaged data. Data were averaged across Test 1 and Test 2 in each task. RaLc, the anode on the right primary motor cortex (M1) and the cathode on the left M1; RcLa, the anode on the left M1 and cathode on the right M1. *p < 0.05.

Because Type III ANOVA revealed a difference in stimulation conditions (RcLa > Sham), a *post hoc* test on a subset of contrast between handgrip tasks was performed on the estimates to confirm the hypothesis that the extent of stimulus effect on unimanual and bimanual movement is different. In the simultaneous bimanual task, bimanual handgrip force for both hands was significantly higher in the RcLa condition than in the Sham condition (left, Sham: 350 ± 76 vs. RcLa: 357 ± 69 N, Estimate = 11 N, SE = 4, *t*_(29.5)_ = 2.501, *p* = 0.018, *d* = 0.10; right, Sham: 370 ± 86 vs. RcLa: 381 ± 82 N, Estimate = 15 N, SE = 5, *t*_(17.3)_ = 2.841, *p* = 0.011, *d* = 0.14). The bimanual left and right handgrip forces were high in 14 and 15 participants, respectively (Figures 2A,C). In the unimanual task, unimanual left handgrip force was significantly higher in the RcLa condition than in the Sham condition (Sham: 357 ± 76 vs. RcLa: 362 ± 71 N, Estimate = 9 N, SE = 4, *t*_(29.9)_ = 2.158, *p* = 0.039, *d* = 0.08). 14 participants exhibited higher unimanual left handgrip force (Figures 2B,D). Unimanual right handgrip force was also significantly higher in the RcLa condition compared with that in the Sham condition (Sham: 379 ± 86 vs. RcLa: 389 ± 80 N, Estimate = 14 N, SE = 5, *t*_(22.4)_ = 2.726, *p* = 0.012, *d* = 0.13). 17 participants exhibited higher unimanual right handgrip force (Figures 2B,D). In the RaLc condition, although a small number of subjects exhibited higher handgrip force on individual observed values, other subjects exhibited lower handgrip force, and the findings were not consistent (Figure 2). For visualization, Figures 2A,B show the average of the observed values across Test 1 and Test 2 for each individual, and Figures 2C,D show a change in handgrip force related to the Sham condition.

### Bilateral Deficit

**Figure 3:**
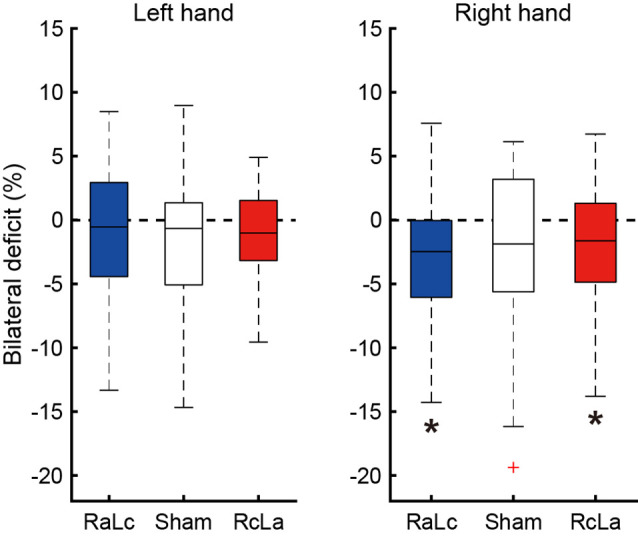
Bilateral deficit (BLD) in handgrip force following the stimulation condition. The horizontal line in the boxes indicates the median, the boxes extend to the 25th and 75th percentiles, the whiskers extend to the extreme values, and the cross represents the outlier. Negative values indicate less bimanual handgrip force compared with that in unimanual handgrip force. RaLc, the anode on the right primary motor cortex (M1) and the cathode on the left M1; RcLa, the anode on the left M1 and cathode on the right M1. *p < 0.05.

BLD, in which the bimanual handgrip force was lower than unimanual force, was confirmed under some stimulation conditions (Figure 3). In the right hand, BLD was observed in the RcLa and RaLc conditions, which indicates that bimanual handgrip force was lower than unimanual handgrip force (RaLc: −3.39 ± 5.31%, *t*_(20)_ = −2.922, *p* = 0.004, *d* = 0.64; RcLa: −2.24 ± 5.59%, *t*_(20)_ = −1.832, *p* = 0.041, *d* = 0.40). For the Sham condition, there was a small effect size and a trend for BLD to occur (Sham: −2.47 ± 6.73%, *t*_(20)_ = −1.679, *p* = 0.055, *d* = 0.37). For the left hand, there were small effect sizes and trends for BLD to occur in the Sham and RcLa conditions (Sham: −1.83 ± 5.63%, *t*_(20)_ = −1.491, *p* = 0.076, *d* = 0.33; RcLa: −1.19 ± 3.79%, *t*_(20)_ = −1.441, *p* = 0.083, *d* = 0.31). For the RaLc condition, BLD was not observed and unimanual and bimanual handgrip force were comparable (RaLc: −0.75 ± 4.92%, *t*_(20)_ = −0.701, *p* = 0.246, *d* = 0.15).

### Adverse Effects and Placebo Effect

Skin sensation, redness, and placebo effects were summarized in Table 2. Although there were no serious adverse effects, some participants reported itching, pain, tingling, warmth, and redness under the electrodes. In response to the question “Do you believe that you received a real or placebo stimulation?, ” half of the subjects reported “I don’t know, ” which indicated no apparent placebo effect.

**Table 2:** Adverse effects and placebo effects.

	RaLc				RcLa				Sham				
	None	Mild	Moderate	Strong	None	Mild	Moderate	Strong	None	Mild	Moderate	Strong	*p*-value
Itching	18	2	1	0	18	3	0	0	18	3	0	0	1
Pain	17	4	0	0	14	7	0	0	17	4	0	0	0.223
Burning	21	0	0	0	21	0	0	0	21	0	0	0	-
Warmth	19	2	0	0	19	2	0	0	18	3	0	0	0.846
Metallic	21	0	0	0	21	0	0	0	21	0	0	0	-
Fatigue	21	0	0	0	21	0	0	0	19	2	0	0	0.135
Alertness	21	0	0	0	20	1	0	0	19	2	0	0	0.368
Tingling	14	7	0	0	17	4	0	0	16	5	0	0	0.311
Redness	16	3	2	0	12	8	1	0	17	3	1	0	0.895
	**Real**	**Placebo**	**I don’t know**		**Real**	**Placebo**	**I don’t know**		**Real**	**Placebo**	**I don’t know**		***p*-value**
Real	7	2	12		6	3	12		7	3	11		0.293

*Values indicate the number of participants. RaLc, the anode on the right primary motor cortex (M1) and the cathode on the left M1; RcLa, the anode on the left M1 and cathode on the right M1*.

## Discussion

The present study sought to elucidate the effects of tDCS of bilateral M1 on unimanual and simultaneous bimanual handgrip strength. Although we expected that the effect of tDCS on bimanual movement would differ from the effect on unimanual movements, greater handgrip strength in the RcLa condition was confirmed to the same extent in the unimanual and bimanual handgrip tasks. We found that bimanual handgrip forces in both hands were higher in the RcLa condition with the anode on left M1 and cathode on right M1 than in the Sham condition. Furthermore, unimanual left and unimanual right handgrip forces were higher in the RcLa condition. These findings suggest that the neuromodulation induced by RcLa tDCS leads to brain states that can produce greater unimanual and bimanual handgrip strength. In addition, these stimulus responses were asymmetrical and were observed in the RcLa condition but not in the RaLc condition.

## Effects of RcLa tDCS on Bimanual Handgrip Strength

To the best of our knowledge, this is the first study to examine the effects of bilateral tDCS on simultaneous bimanual handgrip strength. The bimanual right handgrip force was higher in the RcLa condition with the anode on left M1 and the cathode on right M1 than in the Sham condition. Practically, anodal tDCS of M1 produces facilitation of contralateral limb performance, such as increased motor evoked potential (MEP) amplitude (Nitsche and Paulus, 2000), MVC (Krishnan et al., 2014), and muscle endurance (Cogiamanian et al., 2007), and promotion of motor learning (Fan et al., 2017). Therefore, greater bimanual right handgrip strength in the RcLa condition may be caused by the facilitation of contralateral corticospinal excitability in left M1. In addition to bimanual right handgrip force, bimanual left handgrip force was also higher in the RcLa condition, despite right M1 receiving cathodal stimulation. Assuming a polarity-dependent effect of tDCS (Nitsche and Paulus, 2000), RcLa tDCS may inhibit excitability of contralateral corticospinal pathway from right M1 due to cathodal stimulation. However, the bimanual left handgrip force did not decrease in the RcLa condition; rather, it was higher in this condition. Considering that left M1 received the anodal stimulation in the RcLa condition, it is possible that the facilitation of the ipsilateral motor pathway from left M1 (Ziemann et al., 1999; Ziemann and Hallett, 2001; Lacroix et al., 2004) and/or motor neurons that are active during bimanual movement (Aizawa et al., 1990; Donchin et al., 1998) in the left M1 affected bimanual left handgrip force. However, there is no direct evidence suggesting tDCS-induced facilitation of these motor pathways. On the other hand, recent studies have reported that cathodal tDCS of M1 increases corticospinal excitability regardless of polarity-dependent effects (Batsikadze et al., 2013; Wiethoff et al., 2014), and anodal tDCS of M1 increases bilateral corticospinal excitability regardless of the side stimulated (Rahman et al., 2020). Batsikadze et al. (2013) reported that 1 mA cathodal tDCS of M1 decreases corticospinal excitability, whereas 2 mA cathodal tDCS increases it. In the present study, the current intensity was 1.5 mA, which is stronger than 1 mA, so it may have modulated M1 to excitatory. In any case, we did not assess corticospinal excitability and therefore could not determine which parts of the neural circuits are facilitated by tDCS. However, we found that bimanual handgrip strength was greater in both hands in the RcLa condition. Therefore, our findings suggested that RcLa tDCS leads to brain states that can produce greater simultaneous bimanual handgrip strength.

## Effects of RcLa tDCS on Unimanual Handgrip Strength

Interestingly, the RcLa condition was also associated with greater unimanual handgrip strength, as well as bimanual handgrip strength. Despite the right M1 receiving cathodal stimulation, the unimanual left handgrip force in the RcLa condition was higher than in the Sham condition. A recent study demonstrated that tDCS of bilateral M1 promoted motor learning at both the anodal and cathodal sides in unimanual movement (Waters et al., 2017). Furthermore, the researchers reported increased brain activity during learned unimanual movement (Waters et al., 2017), suggesting polarity-independent neuroplasticity in the tDCS of bilateral M1. This indicates that the motor limb innervated by the M1 receiving cathodal stimulation may also induce an improvement in unimanual movement. Moreover, the unimanual right handgrip force was also higher in the RcLa condition than in the Sham condition. These effects may be associated with the facilitation of contralateral corticospinal excitability in left M1. Bilateral tDCS produces improvement of contralateral unimanual hand function, including precise movement (Vines et al., 2008) and reaction time (Karok et al., 2017). In the current study, we reproduced a previous finding showing improvement of contralateral unimanual hand movement by bilateral tDCS and demonstrated greater force-generating capacity of unimanual right handgrip in the RcLa condition. As mentioned in the previous section, it is difficult to determine which neural circuits the RcLa tDCS facilitated. However, greater unimanual left and unimanual right handgrip strength were observed in the RcLa condition. Therefore, rather than a polarity-dependent effect, it is possible that the RcLa tDCS caused bilateral M1 to produce the brain states generating large motor commands required for unimanual force generation.

## Effects of RaLc tDCS on Handgrip Strength

In contrast to RcLa tDCS, greater unimanual and bimanual handgrip forces were not observed in either hand in the RaLc condition, compared with those in the Sham condition. The current study demonstrated that RcLa and RaLc tDCS induced different changes in handgrip strength, indicating asymmetric stimulus effects in the RcLa and RaLc conditions. However, because there was no significant difference between the RcLa and RaLc conditions, we were unable to draw any conclusions regarding whether the effect depended on the electrode position. Therefore, further studies examining the laterality of the brain and differences in the direction of the current flow of tDCS are needed.

## Bilateral Deficit

In the current study, a trend in BLD for both left and right handgrip was observed in the Sham condition, but this was not significant. Because BLD appears to exhibit high variability in magnitude and existence and appears to be plastic (Škarabot et al., 2016), our results may have been influenced by these effects. Although BLD in right handgrip strength was observed in the RaLc and RcLa conditions, it exhibited no apparent change. Because the greater unimanual and bimanual handgrip strength were induced by RcLa tDCS, no apparent changes in BLD were likely to have occurred. In the RaLc condition, BLD appeared to be enhanced, but it was not possible to determine whether this was due to an increase in unimanual right handgrip strength or a decrease in bimanual right handgrip strength. Conversely, for the left hand, no apparent BLD was observed in the RaLc and RcLa conditions. A comparable increase in both unimanual and bimanual left handgrip strength due to RcLa tDCS may not affect the degree of BLD. Therefore, the correct interpretation of the effect of bilateral tDCS on BLD in handgrip strength remains unclear.

## tDCS Applications in Sports Performance and Neurorehabilitation

tDCS-induced improvements in sports performance have been reported in endurance performance in cycling (Angius et al., 2018) and unilateral single–joint movement (Cogiamanian et al., 2007), and jumping performance (Lattari et al., 2020). However, both positive (Tanaka et al., 2009; Krishnan et al., 2014) and negative (Cogiamanian et al., 2007) effects of tDCS on MVC strength have been reported. The current study revealed that unimanual and bimanual handgrip strength in both hands were greater in the RcLa condition, although the effect size was relatively small (Estimate = 9–15 N; *d* = 0.08–0.14). However, our findings will have practical importance in sports, where 0.01 s or 1 cm can make or break a game. In addition, clinical studies have reported the efficacy of tDCS of bilateral M1 in stroke patients (Lindenberg et al., 2010). The current study revealed greater motor performance in healthy subjects, providing evidence to inform the application of tDCS in the treatment and neurorehabilitation of patients with motor dysfunction.

## Limitations

A limitation of the current study was the absence of pre/post measurements. In the current study, considering the fatigue effects caused by repeating the maximal handgrip test, the experimental procedure was designed for comparison between post-stimulation handgrip strength and the Sham condition. However, pre/post design experiments are needed to investigate whether tDCS clearly improves the handgrip strength in each stimulation condition. As another limitation, anodal and cathodal effects could not be separated because we did not compare them with stimulation effects for a single brain region. Future studies should compare these effects with high definition tDCS. In the current study, because we did not assess neurophysiological parameters, such as MEP amplitude and IHI, it was difficult to identify the excitability of the corticospinal motor pathway and transcallosal pathway. It is necessary to evaluate the corticospinal excitability to assess the different neuromodulation depending on the current intensity or the direction of current flow. Furthermore, although handgrip is a relatively simple experimental task, it involves both agonist (flexor) and antagonist (extensor) muscle contractions (Hoozemans and van Dieän, 2005). No stimulus effect was seen in the EMG of the flexor digitorum superficialis in the current study. Therefore, caution is necessary in interpreting the handgrip results because interference of motor commands may have occurred. In addition, although our results revealed the difference between conditions in terms of group data, individual data exhibited variability in response to tDCS for the handgrip strength. Future studies are needed to clarify the inter- (Wiethoff et al., 2014) and intra-individual variability (Chew et al., 2015) of tDCS effects in order to explore its application in sports. Taken together, because the neural circuits involved in the experimental task and stimulation parameters differ from study to study, studies examining the effects of tDCS in various experimental paradigms should be reported.

## Conclusions

We investigated the effects of tDCS on bilateral M1 on unimanual and simultaneous bimanual movements. The current findings revealed greater unimanual and bimanual handgrip strength in both hands after RcLa tDCS with the anode on left M1 and the cathode on right M1. These findings suggest that the neuromodulation induced by RcLa tDCS leads to brain states that can produce greater unimanual and bimanual force generation.

## Data Availability Statement

The data that support the findings of this study are available from the corresponding author, YA, with permission from our ethical committee.

## Ethics Statement

The studies involving human participants were reviewed and approved by the Human Subjects Committee at Chukyo University Graduate School of Health and Sport Sciences. The patients/participants provided their written informed consent to participate in this study.

## Author Contributions

MH and YA designed the study, analyzed the measured data, and wrote the article. MH performed the measurements. All authors contributed to the article and approved the submitted version.

## Conflict of Interest

The authors declare that the research was conducted in the absence of any commercial or financial relationships that could be construed as a potential conflict of interest.
